# A periodic split attractor reconstruction method facilitates cardiovascular signal diagnoses and obstructive sleep apnea syndrome monitoring

**DOI:** 10.1016/j.heliyon.2024.e35623

**Published:** 2024-08-03

**Authors:** Ze Zhang, Kayo Hirose, Katsunori Yamada, Daisuke Sato, Kanji Uchida, Shinjiro Umezu

**Affiliations:** a Graduate School of Creative Science and Engineering, Department of Modern Mechanical Engineering, Waseda University, 3-4-1 Okubo, Shinjuku-ku, Tokyo, 169-8555, Japan; b Department of Anesthesiology and Pain Relief Center, The University of Tokyo Hospital, 7-3-1 Hongo, Bunkyo-ku, Tokyo, 113-8655, Japan; c Faculty of Economics, Kindai University, 228-3 Shin-Kami-Kosaka, Higashi-Osaka, 577-0813, Japan; d Department of Pharmacology, University of California, Davis, Genome Building Rm3503, Davis, CA, 95616–8636, USA

**Keywords:** ECG, Periodic split attractor reconstruction, Cardiac diseases, OSAS, Intelligent diagnosis

## Abstract

Electrocardiogram (ECG) is a powerful tool to detect cardiovascular diseases (CVDs) and health conditions. We proposed a new method for evaluating ECG for efficient medical diagnosis in daily life. By splitting the signal according to the cardiac activity cycle, the periodic split attractor reconstruction (PSAR) method is proposed with time embedding, including three types of splitting methods to show its chaotic domain characteristics. We merged the CVDs dataset and the obstructive sleep apnea syndrome (OSAS) first-lead ECG signal dataset to validate the performance of PSAR for diagnosis and health monitoring using PSAR density maps as SE-ResNet input features. PSAR under 3 split methods showed different sensitivities for different CVDs. While in OSAS monitoring, PSAR showed good ability to recognize sleep abnormalities.

## 1 Introduction

Cardiovascular diseases (CVDs) are one of the two main causes of death, which have been a common threat to all mankind [1,2]. The prevalence of obstructive sleep apnea syndrome (OSAS), which is associated with cardiovascular disease, is as high as 40 %–80 % [3], and the prevalence of the disease itself is about 14 % in men and 5 % in women [4]. The aggravation and accumulation of co-morbidities further contributes to the increase in mortality [5], so the impact of the association between CVDs and OSAS has been emphasized and evaluated [6,7].

Patients who are not aware of OSAS are at increased risk for CVDs, as well as stroke, cognitive decline, depression, and premature death [5], which also significantly increases the burden on the healthcare system [8,9]. Therefore, it would be valuable to develop an effective methodology for both diagnosis and health detection of CVDs and OSAS.

The electrocardiogram (ECG) contains a wealth of information about the activity of the human heart, which reflects the heart health of physiological activities. Researchers conducted their studies on ECG based rapid diagnosis methods of heart diseases, which are used for cardiac status monitoring, and high precision monitoring in 12 and 18-lead has been widely studied in the clinic, while single-lead is also used in daily health monitoring and wearable smart devices [[10], [11], [12]]. The technical criterion for the diagnosis of OSAS is polysomnography (PSG) [13], which requires prolonged monitoring of airflow, blood saturation, and multichannel signaling such as ECG and manual labeling in order to accurately diagnose the disease. Although researchers have attempted to monitor OSAS by a single signal, including blood oxygen saturation [14], photoplethysmography (PPG) [15] and so on, to reduce the monitoring cost, the influence of CVDs has not been taken into account. We are committed to establishing a scenario that applies to daily life health monitoring that does not rely on complex instrumentation operations and specifications for simultaneous diagnosis of CVDs and OSAS monitoring. Therefore, we note that the I-lead ECG signal can serve as a key bridge connecting the diagnostic techniques for both types of diseases [16].

In this paper, we propose a periodic split attractor reconstruction (PSAR) method and validate the diagnostic accuracy of this method as a machine learning feature input using SE-ResNet. We reconstructed the I-lead dataset, which is easy to manipulate in daily life, based on the cardiac physiological cycle as a time-embedding parameter, and analyzed the performance of PSAR performance under R–R split, Q-R-S split, and S-TP-Q split for this signal. The results shows that the method does not depend on a specific ECG signal acquisition duration. Its diagnostic ability in 6 kinds of CVDs with 3 split methods showed the diagnostic sensitivity for different diseases. In OSAS monitoring, F1-score of 100 % (OSAS), Specificity of 100 % (OSAS) was achieved for all three split methods. The results show that the proposed PSAR method for ECG signals can be used as a visual and interpretable feature, which combined with machine learning algorithms can achieve high-precision and fast diagnosis of cardiovascular diseases, and also has the ability to diagnose non-standard signals.

## 2 Previous research on ECG and chaos theory

ECG components are rich and ample, which include information such as clinical frequencies, heart rate measurement, R–R interval measurement, spectral components, non-linearity, trajectory identification, and amplitudes. Understanding and processing of the corresponding features to represent the subtle changes caused by the disease is the main purpose of heart monitoring [17,18]. Previous researchers have focused on the perspective of the time domain [19,20] and the frequency domain [21,22] in quantifying and explaining the variability of cardiovascular signals. In this study, we rather focus on nonlinear dynamics in ECG data: since the study of nonlinear dynamics has been proved to be an excellent method to understand and quantify many biological and physiological events in the human body [23,24], we address this method to the monitoring of CVDs and OSAS.

One way of addressing nonlinear dynamics in time series data is transforming one-dimensional data into multi-dimensional phase space. So, the dynamics of ECG data in the time domain can be mapped into the phase space. The geometric features are captured on different sub-dimensional phase planes of the data, such as Euclidean eigenvalue and central point distribution, which would indicate key biomarkers of different ECG states [25]. The chaotic eye coordinates of specific ECG signals are obtained by transforming ECG data into chaotic dynamic error distribution maps through a master-slave chaotic system, which can also achieve efficient signal classification [26]. By using chaotic signals to functionally represent ECG signal sets, the ambiguity associated with the trajectory estimation and with recurrence period entropy indexes is reduced, and the transitions in rhythmic behavior become more prominent for analysis [27].

With the continuous study of the chaotic model, we are not satisfied with just confirming whether there is an arrhythmia in ECG signal, but establishing a method to diagnose more specific CVDs quickly and effectively. Starting from the one-dimensional time series describing the behavior of the system, the time-delay method is used to construct the attractor of the dynamic system in the multi-dimensional state space [28]. The method of attractor reconstruction has been successfully applied to biological signal processing including ECG [[29], [30], [31], [32]], blood pressure signal [[33], [34], [35]], photoplethysmography (PPG) [36], and respiration [37,38]. With the advantage that biological signals usually regarded as stretching along the time axis can be represented in bounded reconstructed phase space, a new method for analyzing blood pressure data is developed and analyzed, so it can detect the change of waveform shape that cannot be detected by heart rate variability method [34,39]. Furthermore, attractor reconstruction is being applied as a method to efficiently observe the biomarkers [40,41], and progressively deployed in state detection scenarios for a variety of organisms including horses [32], rats, and so on.

In the diagnosis of abnormalities by ECG signals, including CVDs and OSAS, the complexity of the attractors makes it difficult to distinguish between signals, despite preprocessing to remove errors such as noise and drift. Therefore, we propose period split attractor reconstruction to enhance signal coherence for extraction into interpretable features by pinpointing target split. It is applied to easily acquired first-lead ECG signals to enhance the utility of wearable and portable cardiovascular monitoring devices.

## 3 Methodology

### 3.1 Overview

**Fig. 1:**
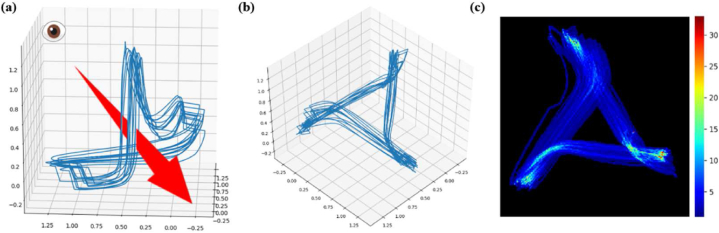
Attractor reconstruction. (a) Attractor reconstruction method based on cardiac physiological cycle split using τ = (Split period)/3 [42], (b) A projection of the trajectory onto the plane orthogonal to the x = y = z axis, (c) The trajectory turned into a density map.

Our research objective is to analyze and diagnose the health status of patients by using rich ECG waveform information. Firstly, we provide a technical roadmap for the diagnostic method, followed by a detailed introduction of each step（see Fig. 1).

### 3.2 Database

Two types of data were used in this study, the first for CVDs diagnosis and the second for health status monitoring. Both types of datasets explicitly recorded lead information and were labeled by physicians for disease and specific signal locations.

For CVDs diagnosis, the performance of the proposed diagnostic analysis method was evaluated on two part of databases with the 500 Hz sampling rate, a 12-induced ECG dataset for arrhythmia research created by Zheng et al. in 2020 (Database1) [43] and a large-scale multi-label 12-lead ECG database with standardized diagnostic statements (Database2) [44]. In this study, I-lead was chosen for the rapid diagnostic test based on proposed method. We extracted 6 disease signals from two datasets to form a sample homogeneous dataset for the validation of the proposed method in this paper, as shown in Table 1.

**Table 1:** Rhythm information and original data volume.

Full Name	Acronym Name	Original Data Count	
		Database1 [43]	Database2 [44]
Normal Sinus Rhythm	NSR	0	13905
Sinus Bradycardia	SB	3889	2711
Atrial Fibrillation	AFIB	1780	675
Atrial Flutter	AF	445	99
Sinus Tachycardia	ST	1568	725
Right Bundle-branch Block	RBBB	0	710

For sleep state monitoring data, we chose the obstructive sleep apnea syndrome (OSAS) dataset of PSG recordings that had been manually standardized by a medical professional [45]. Separating the I-lead ECG data, the final 145,440s ECG data, which was sampled at 80 Hz.

### 3.3 Pre-processing

In ECG data acquisition, power line interference, electrode contact noise, motion artifacts, muscle contraction, and other noise can interfere with signal analysis and CVDs diagnosis. In the preprocessing step, we removed the noise and baseline drift, and located the peak coordinate in the ECG signal, so as to construct the reconstruction attractor method in Section 3.4.

#### 3.3.1 Discrete wavelet transform

In the ECG, the meaningful frequency band is determined to some extent, and the data in the low frequency band of 0.05 Hz and the high frequency band of 100 Hz or more is regarded as noise [46]. Therefore, noise reduction using frequency analysis is required. In this paper, discrete wavelet transform (DWT) is used for frequency separation and also for peak signal localization.

The signal of the function f(x) is sampled with N values $C_{0},C_{1},\ldots ,C_{N-1}$ and decomposed into j frequency bands, $d_{k}$ is detail coefficients, which is shown in (1), (2).

(1) $$f\left(x\right)=\sum_{j=1}^{n}\;\sum_{k=0}^{N/2^{j}-1}\;d_{k}^{\left(j\right)}\psi_{k}^{\left(j\right)}\left(x\right)+c_{0}^{\left(n\right)}$$

(2) $$d_{k}=\frac{c_{2k}^{\left(j-1\right)}-c_{2k+1}^{\left(j-1\right)}}{2}$$

**Fig. 2:**
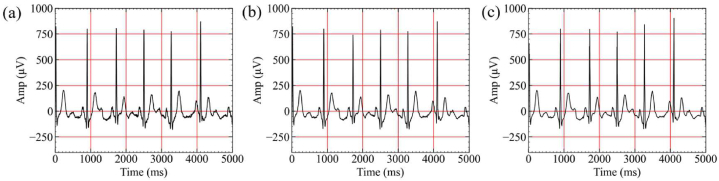
ECG signal preprocessing results. (a) Raw ECG containing noise, (b) An ECG after noise reduction, (c) An ECG containing baseline wandering.

The $\psi_{k}$ is the mother wavelet function, Daubechies (db6) was used for wavelets [47]. The noise is removed by removing the signal in the low frequency (A10) and high frequency (D1, D2) bands from the frequency characteristic (A10, D1 to D10) obtained by the wavelet transform, the denoised result was presented in Fig. 2(b) and 10-level decomposition signal is shown in Supplementary Material Fig. S3.

#### 3.3.2 LOESS curve fitting

The local polynomial regression smoother (LOESS) [48] was used to remove baseline wandering. The smoother was fitted using weighted least squares, where the weighting function assigns maximum weight to the data point closest to the estimation point and minimum weight to the data point furthest away. We subtracted the LOESS estimated trend on D10, D9, and D8 (obtained from the 3.3.1) to clear the effect of baseline wandering, shown as Fig. 2(c).

#### 3.3.3 Wave group extraction in the I-lead

ECG signal have peaks called PQRST-waves that appear periodically, cardiac transmission information for each peak is included [49]. In existing studies, researchers tend to use intact ECG signals, but we noticed that a portion of the characteristic signal is masked in the attractor density on the way. In this study, we took advantage of the fact that the ECG features of each disease manifested in different locations to construct a new attractor based on the peak cycle splitting signal. By using the sliding window method [47], the curvature changes within different time intervals are extracted to obtain the peak values of R, Q, S, T, and P. The detail is shown in supplementary material section S.1.

### 3.4 Attractor reconstruction based on split cardiac cycle

**Fig. 3:**
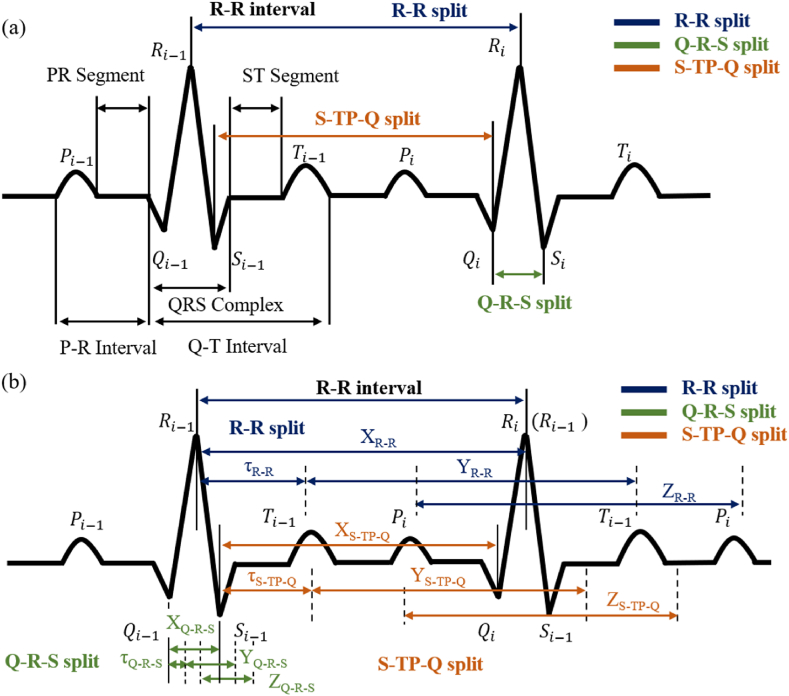
The ECG waveform and split method in I-lead that presents a normal cardiac cycle. (a)The ECG waveform and split that presents a normal cardiac cycle. (b) Proposed periodic spilt attractor reconstruction method based on ECG signal. The blue, green and red lines represent R–R split, Q-R-S split and S-TP-Q split method respectively. (For interpretation of the references to colour in this figure legend, the reader is referred to the Web version of this article.)

Cardiac cycle, which is usually manifested as a series of waveforms on the ECG signal, is shown in Fig. 3(a). As a time-domain signal, based on Takens [28], constructing attractor via time delay embedding, which is represented by Eq. (3) and the time series data after time *n* is $y_{n}$.

(3) $$v\left(n\right)=\left(y_{n},y_{n+\tau },\ldots ,y_{n+\left(m-1\right)\tau }\right)$$

It is worth noting that for periodic signals, researchers usually use 1/3 of the mean value of the period as the time delay *τ* [35,50,51], and according to the analysis of the attractor feature performance properties (shown in section 4.1), the effect of attractor reconstruction based on the mean value as the τ does not perform consistently on different periods, and its cumulative error increases further with the growth of the signal time. Therefore, we propose a time-embedded reconstruction method based on cardiac cycles split, which is periodic split attractor reconstruction (PSAR). Similarly, reconstructed in an *m*-dimensional ‘phase space’ from single lead signal by using a vector of delay coordinates, x(*t*) is shown in Eq. (4).

(4) $$\left[x\left(t_{p1}\right),x\left(t_{p1}-\tau_{p1}\right),x\left(t_{p1}-2\tau_{p1}\right),\ldots ,x\left(t_{p1}-\left(m-1\right)\tau_{p1}\right),\ldots ,x\left(t_{pi}\right),x\left(t_{pi}-\tau_{pi}\right),x\left(t_{pi}-2\tau_{pi}\right),\ldots ,x\left(t_{pi}-\left(m-1\right)\tau_{pi}\right)\right]$$

Where, pi is period id, $\tau_{pi}$ is delay parameter corresponding to the splitting cycle, *m* is the embedding dimension, *m* = 3.

The key to the split, is that each split data segment is separately embedded in time and re-spliced, shown as Fig. 3(b). This ensures the integrity of the expectation signal and guarantees the consistency of the different eigenwaves in the attractor performance.

Attractor reconstruction based on the split cardiac cycle is based on peak detection (Section 3.3), where the complete ECG signal is split through the cycle before being re-spliced (which is shown in Fig. 3 (b)), and the attractor reconstruction is performed according to the corresponding time delay parameter within each split cycle. In Fig. 3, the blue line representing the R–R split is illustrated, where Fig. 3 (a) shows the normal cardiac activity signal sequence, and the split cardiac activity signal sequence is shown in Fig. 3 (b). The $X_{R-R}$ range is from $R_{i-1}$ to $R_{i}$, and the $Y_{R-R}$ range is in the next sampling point of $R_{i}$ when the data is re-continued for one tau from $R_{i-1}$, and similarly, the z-coordinate $Z_{R-R}$ is the same.

The reconstructed attractor can gather the information of a period of time into an image. As the amount of data increases, the data points in the image will overlap with each other. Therefore, we use projection density as a feature to map the patient's state. However, since the amplitude of different signal peaks can vary greatly, directly, the R-wave voltage usually fluctuates between 0.5 mV and 2.0 mV, while the P-wave is usually less than 0.25 mV. Some of the characteristic signals are not easy to capture accurately in the attractor density maps, and it is therefore necessary to split the signal in order to capture the potential features.

**Fig. 4:**
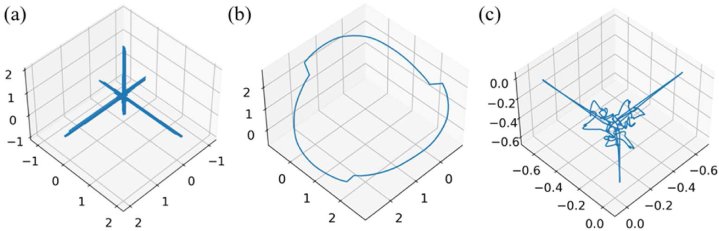
PSAR of the NSR signal in different split method. (a) R–R split, (b) Q-R-S split, (c) S-TQ-P split.

For split signal, we adopted three split methods, which is shown in Fig. 3 (b). The first method is to extract two adjacent *R*-peaks, including $R_{i-1}$-peak, $S_{i-1}$-wave, $T_{i-1}$-wave, $P_{i}$-wave, $Q_{i}$-wave, and $R_{i}$-peak. The second method is to extract $Q_{i}$-peak to $S_{i}$-peak, which includes the $R_{i}$-wave, defined as Q-R-S split. The third method is the $S_{i-1}$-peak from the $Q_{i}$-peak to the next cycle, includes $T_{i-1}$-wave, $P_{i}$-wave, defined as S-TP-Q split. We use a section of NSR signal to illustrate the reconstruction result of its split attractor, as shown in Fig. 4.

### 3.5 Classifier model

#### 3.5.1 SE-ResNet

We choose the general model as the feature validity detection tool of the reconstruction split attractor. Considering the changes in spatial coordinates, we choose the attention mechanism Squeeze and Excitation (SE) to focus on the changes between channels [[52], [53], [54]]. Divide dataset according to two standards: classify data into different time periods, and test the classification effect of different time periods. Test the classification performance of different data split methods under controlled time length conditions.

#### 3.5.2 Performance metrics

(5) $$Accuracy=\frac{TP+TN}{TP+FP+FN+TN}$$

(6) $$Precision=\frac{TP}{TP+FP}$$

(7) $$Recall=\frac{TP}{TP+FN}$$

(8) $$Specificity=\frac{TN}{TN+FP}$$

(9) $$F_{1}=\frac{2*\;Precision\;*\;Recall\;}{\;Precision+Recall\;}$$

The counts of true positives *TP*, true negatives *TN*, false positives *FP*, and false negatives *FN* are represented via a confusion matrix. Accuracy, precision, recall, specificity and F1-scores are calculated and we use these scores for evaluation.

## 4 Results & discussion

### 4.1 PSAR with delay time

For proposed attractor reconstruction method based on split cardiac cycle, we use the ideal signal to demonstrate the effect of reconstruction parameter time delay on ECG signals.

#### 4.1.1 Single cycle signal with different delay τ

**Fig. 5:**
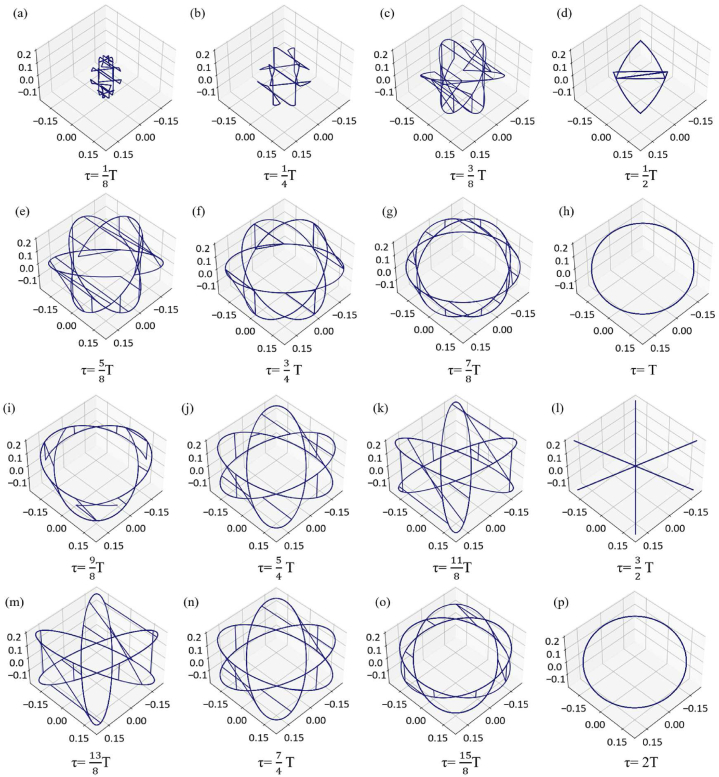
PSAR of same period signal with different delay τ.

We conduct single cycle sine function simulation according to the ECG signal cycle and R-peak amplitude of I-lead, subdivide the periods [0,2T] into 16 states, and its split attractor reconstruction performance is shown in Fig. 5, the delay results of 16 equal periods are shown in Fig. S1 (period [0-T]) and Fig. S2 (period [T-2T]), respectively. The signal is shown in Eq. (10).

(10) $$y=0.2∙\sin\left(\pi \frac{x}{400}\right)$$

The cycle period of each sine function can be approximated as a QRS-wave group, as shown in Fig. 3. When the parameter is very small compared to the period, as shown in Fig. 5 (a) (b) (c) (d), it is difficult to reflect the characteristics and converge in a smaller space, which helps us filter out a portion of high-frequency (relative to the target wave group) data in a certain sense. However, it can also result in the loss of some of the unmarked or unrecognized features. We can obtain a complete arc for τ = T and τ = 2T, Fig. 5 (h)(p). When this parameter fluctuates near the complete period, it tends to change into a centrosymmetric pattern, as shown in Fig. 5 (g) (i) (j) (o). This central symmetry feature is caused by the peak coordinate rotation caused by the parameter period, which will be further explained in the next.

#### 4.1.2 Multi cycle signal with different delay τ

In the I-lead, there is currently a P-QRS-T complex. Therefore, we further analyze the reconstruction characteristics of attractor under complex conditions by adjusting the period and peak amplitude of each split through piecewise sine function.

**Fig. 6:**
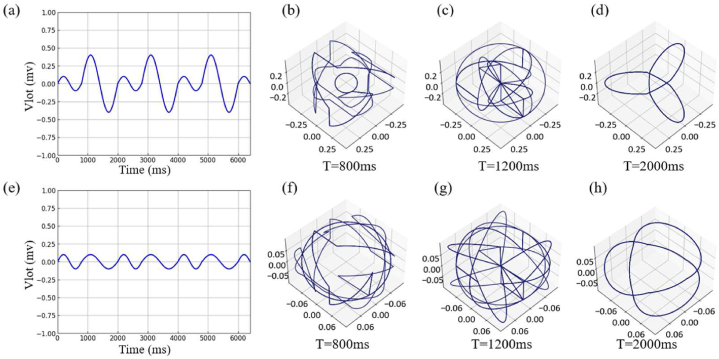
Mixed periodic signal. (a) The waveform Eq. (11) of two spliced sinusoidal signals with different periods and different amplitudes, in which $T_{1}=800\text{ms}$, $T_{2}=2000\text{ms}$, $A_{1}=0.1\text{mv}$ and $A_{2}=0.4\text{mv}$ (b) PSAR under periodic split T=800ms. (c) PSAR under periodic split T=1200ms. (d) PSAR under periodic split T=2000ms. (e) The waveform Eq. (12) of two spliced sinusoidal signals with different periods and same amplitudes, in which $T_{1}=800\text{ms}$, $T_{2}=2000\text{ms}$ and $A_{1}=A_{2}=0.1\text{mv}$. (f) PSAR with periodic split T=800ms. (f) PSAR with periodic split T=800ms. (c) PSAR with periodic split T=1200ms. (g) PSAR with periodic split T=2000ms.

Fig. 6 (a) represents signals with different periods and peak amplitudes in each split, which is shown in Eq. (11). In (b), we can observe that there is a complete arc in the center, which is the phase space feature of the first split with a period of 800 ms. When entering the part with a period of 1200 ms, the signal is symmetrically spread out. Similarly, (c) since the delay parameter and periodic signal are not multiples, the reconstructed attractor shows a central symmetry under both amplitudes. (d) Since all signals are within the same cycle, the delay can completely divide all peaks, resulting in a complete central symmetric arc. Therefore, we choose the delay parameter to use periodic split as the window of each reconstruction attractor for separate reconstruction. Although this will cause the interval information to be adjacent signals in the physiological interval, it does not affect the validity of the feature description of the reconstructed attractor.

(11) $$\left\{ \begin{array}{c} y=0.1\cdot \sin\left(\frac{\pi x}{400}\right)\;x\in i*\left(0,800\right),i=0,2,4\ldots \;\\ y=0.4\cdot \sin\left(\frac{\pi x}{600}\right)\;x\in j*\left(800,2000\right),j=1,3,5\ldots \end{array}\right.$$

(12) $$\left\{ \begin{array}{c} y=0.1\cdot \sin\left(\frac{\pi x}{400}\right)\;x\in i*\left(0,800\right),i=0,2,4\ldots \;\\ y=0.1\cdot \sin\left(\frac{\pi x}{600}\right)\;x\in j*\left(800,2000\right),j=1,3,5\ldots \end{array}\right.$$

Fig. 6 (e) represents signals with different periods and same peak amplitudes in each split, which is shown in Eq. (12). It is not difficult to see that **(f) (g) (h)** has the same trend as **(b) (c) (d)**, presenting the effect of feature confusion at the same peak amplitude. However, in ECG signals, each peak amplitude is not at the same level under normal circumstances, so its confusion can be used as an effective feature for state interpretation.

#### 4.1.3 Baseline drift

Baseline drift is a common error in the ECG signal acquisition process. When the reconstructed attractor is projected on the (1, 1, 1) vertical plane, its normal vector becomes (u, v, w) as shown in Eq. (13), and the spatial characteristics of the reconstructed attractor will not be affected when it is superimposed on the time-domain signal with a constant value.

(13) $$u=\frac{1}{\sqrt{2}}\left(x+z\right),v=-\frac{1}{\sqrt{3}}\left(x-y+z\right),w=-\frac{1}{\sqrt{6}}\left(x+2y-z\right)$$

**Fig. 7:**
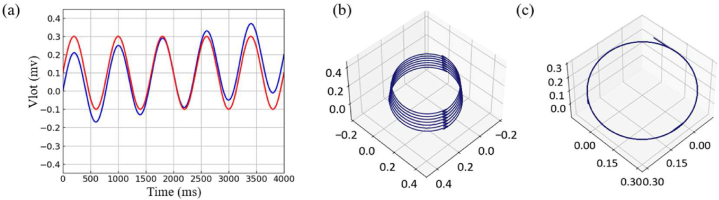
Signal under baseline drift. (a) Waveforms to simulate superimposed signal drift, the blue line represents a superimposed constant signal (Eq. (14)) and the red line represents a superimposed positively proportional signal (Eq. (15)). (b) PSAR with positively proportional signal. (c) PSAR with constant signal. (For interpretation of the references to colour in this figure legend, the reader is referred to the Web version of this article.)

When the red dots and lines are drawn in Fig. 7 (a), as shown in Eq. (14), the signal with a constant superimposed on it will be translated in space, but it will not cause any changes in the features, as shown in Fig. 7 (b). Aston also provided proof in the analysis method of HRV signals.

(14) $$y=0.2\;\sin\left(\pi \frac{x}{400}\right)+0.1$$

However, we will also find that when the superimposed signal increases linearly, the blue dotted line in Fig. 7 (a), as shown in Eq. (15), will cause the spatial features of each cycle to overlap with each other, which is the situation where the curve in the image becomes thicker, as shown in Fig. 7 (b). Therefore, depending on the basic principle of attractor reconstruction, the elimination of baseline drift is limited, and it still needs to be corrected by the baseline drift algorithm. In this paper, the wavelet transform method is selected.

(15) $$y=0.2\;\sin\left(\pi \frac{x}{400}\right)+0.00005x$$

### 4.2 General properties of PSAR

PSAR is directly oriented to time life signals with strong correlation of cycles, in contrast to time-embedded attractor reconstruction [[55], [56], [57], [58]] as well as symmetric projection attractor reconstruction [59,60], whose fundamental properties within cycles remain consistent with the above methods. A comparison of the main properties is shown in Table 2. For attractor reconstruction, the study centers on a gradual shift from the analysis of the parameters of the embedding dimension and delay time for information extraction to the analysis of general properties.

**Table 2:** Comparison of different attractor reconstruction methods.

Method	General properties						Reference
	Original signal	Embedding dimension	Delay coordinates	Symmetry	2D projections	Invariance of time rescaling	
Attractor reconstruction	Continuous.	N ≥ 3	By algorithm design	–	–	Yes	[[55], [56], [57], [58]]
Symmetric projection attractor reconstruction	Continuous, periodic signal with period T.	N = 3; N＞3.	τ = T/N	Z_n_ symmetry	Yes	Yes	[59,60]
Proposed PSAR	Periodically split and reconstructed as a continuous signal.	N = 3	$\tau_{pi}$ = $T_{pi}$/N	Z_3_ symmetry in split period	Yes	Invariance in split period	–

T and $T_{pi}$ represent the signal period and split period, respectively; N represents the embedding dimension; τ and $\tau_{pi}$ represent the delay coordinate and split period delay coordinate, respectively.

As shown in Section 4.1, the complex morphology of the ideal signal changes under different delay parameters, the reasonable formulation of the delay time has an important impact on the accurate extraction of feature information, so the various types of analysis methods for specific signals in order to obtain the optimal delay time is a solution idea. And the ECG signal used directly in this study has a significant period, which is due to the signal being generated by the potential difference changes of the heart work. The consistency of the reconstructed attractor in time scale scaling is reflected in both previous studies and this study Section 4.1.1, therefore, under the uniform embedding dimension (N = 3), The cycle split signal still retains the original signal characteristics. More importantly, the intra-cycle signals respond to the extracted physiological processes inside rather than adjacent to each other, which reduces the complexity of the system to a certain extent, shown as Section 4.1.2. Although the projection overlap between cycles is not affected by the z-axis after projection transformation, the drift of the baseline still exists after cycle splitting, so the operation to remove the baseline drift is still necessary.

Overall, the proposed PSAR can effectively realize the rational planning of the embedding time, and the periodic signal has a direct correlation to the life cycle instead of the non-interpretability of the traditional timing signal. The feature consistency of the attractor reconstruction method is achieved at the cost of less computational effort.

### 4.3 Characterization of CVDs

**Fig. 8:**
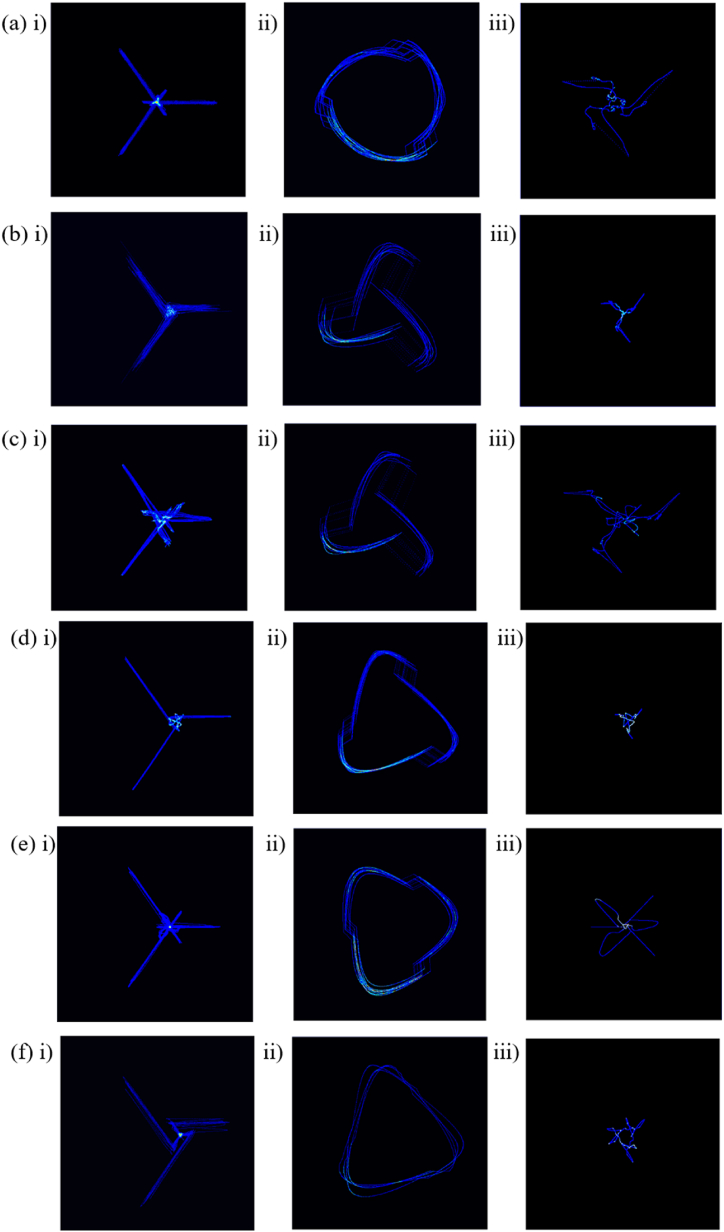
PSAR density map. (a) NSR, (b) AFIB, (c) AF, (d) ST, (e)SB, (f)RBBB. In each group, i) R–R split, ii) QRS split, iii) STPQ split.

After data preprocessing, the attractor image dataset is constructed according to the periodic attractor reconstruction method proposed in this study for the types of CVDs included in the dataset. The PSAR results for R–R split, Q-R-S split, and S-TP-Q split are shown as Fig. 8. It is important to note that since the signal recordings and attractor durations often do not overlap exactly, especially as the length of the used signals increases, the performance of certain features in the dataset is not directly observable to the naked eye. Here we choose as examples the signals corresponding to high frequency of occurrence in the constructed CVDs PSAR dataset.

In Fig. 8, the attractor reconstruction density maps are shown for each of the seven categories of CVDs in the selected dataset, with each group i) ii) iii) corresponding to each of the three types of split methods. For the R–R split, the overall signal signature presents a centrosymmetric three-vertex shape. We use NSR as a benchmark, its signal is stable and therefore shows good signal consistency, when the signal peak shows different point changes, its response to the corresponding vertex position on the feature will change relatively, for example, Fig. 8 (b)i), (c)i) the ratio of its Q-peak, S-peak and R-peak changes.

The center of symmetry part of the response has a smaller amplitude signal, but for the R-peak, it tends to be easily overlooked, including Fig. 8 (c)i), (d)i), (e)i) and (f)i), so Q-R-S split and S-TP-Q split methods can amplify this part of the feature. In the Q-R-S split, the NSR signal is nearly circular despite the fact that three transitions can be observed, whereas the other CVDs signals exhibit features in the form of triangles, and the overall morphology of the CVDs tends to be similar because they reflect only the features of the QRS complex, with AFIB and AF exhibiting similar more diffuse triangles in the tails as in Fig. 8 (b)ii) and (c)ii).

As described in the methodology for S-TP-Q split, not all wave peaks will be stably captured and detected due to the different forms of manifestation waves in some CVDs, so this part of the extraction will include information that can significantly describe the physiological characteristics of the population. However, this part of the reconstruction characterizes and has weak consistency, and often has low consistency between the same dataset and different CVDs datasets. Among them, Fig. 8 (d)iii) (f)ii) corresponds to features with better consistency, corresponding to the ST and RBBB. Methods for further detailed diagnosis of specific diseases, despite their limited role in generalization.

**Fig. 9:**
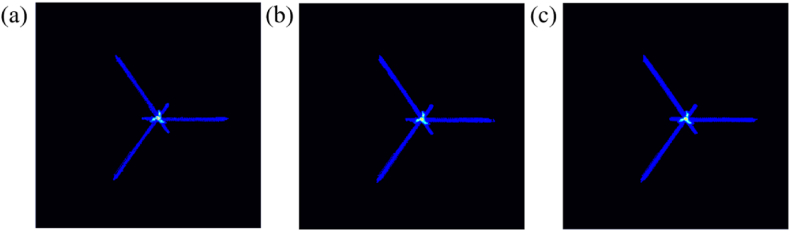
PSAR density map for different time long signals. (a)time = 10s, (b)time = 20s, (c)time = 30s.

Different signal extraction durations are used for data in the dataset for PSAR. This section takes the NSR signal as an example. Under the same pre-processing and PSAR methods, although signal fluctuations will cause broadening of the density map signal characteristics, the NSR signal results show good consistency, which is shown in Fig. 9.

### 4.4 Diagnosis of I-lead CVDs

The classification performance of the model was determined by the accuracy, precision, recall, specificity and F1 scores results. The F1 score is the harmonic mean of precision and recall. It is a more effective criterion for model evaluation than accuracy if the ratio between the data types indicates a strong difference, which is the case for the ECG signal dataset we used in this study.

We evaluated the diagnostic performance of PSAR at different acquisition signal durations. Since the raw data had been segmented into segments by the physicians, we made a classification comparison between NSR and AFIB, two types of diseases that still have a long duration. The effect of duration was evaluated using R–R segmentation, and the 5-fold cross-validation results are shown in Table 3.

**Table 3:** The diagnosis results of PSAR of I-lead ECG under different signal duration in the 5-fold cross validation.

Signal Duration	Evaluation			
	F1	Precision	Recall	Specificity
**5s**	**0.934**	**0.92**	**0.94**	**0.953**
**10s**	**0.945**	**0.936**	**0.943**	**0.96**
**30s**	**0.949**	**0.933**	**0.947**	**0.957**
**Mix**	**0.94**	**0.93**	**0.942**	**0.956**

In Table S1, we tested the diagnosis time of the attractor dataset model built. The CPU calculation time is less than 200 ms, and the GPU calculation time is less than 50 ms. Compared with manual diagnosis, the diagnosis speed is greatly improved.

Table 4 shows the classification result under different split method, among which R–R split generally has higher accuracy. However, in the detection of RBBB, Q-R-S split performs better, SB and ST show better classification performance in the S-TP-Q split. Considering the differences in the performance of different disease types in ECG signals, we can consider assigning weights to different disease types in the analysis to improve the overall detection effect. Diagnostic accuracy confusion matrix of PSAR for CVDs of I-lead ECG signals are shown in Supplementary Material in Fig. S4.

**Table 4:** Comparison of PSAR for I-lead ECG under different splitting methods in 5-fold cross validation.

Signal	R–R Split		Q-R-S Split		S-TP-Q Split	
	Specificity	F1	Specificity	F1	Specificity	F1
**Normal**	**1.0**	**1.0**	**1.0**	**1.0**	**0.97**	**0.944**
**AFIB**	**0.931**	**0.914**	**0.919**	**0.91**	**0.966**	**0.81**
**AF**	**0.945**	**0.903**	**0.942**	**0.896**	**0.978**	**0.841**
**ST**	**0.941**	**0.92**	**0.955**	**0.906**	**0.946**	**0.935**
**SB**	**0.962**	**0.93**	**0.965**	**0.91**	**0.963**	**0.925**
**RBBB**	**0.998**	**0.91**	**0.973**	**0.927**	**0.966**	**0.89**

The method proposed in this article was compared with other studies on the diagnosis of CVDs through ECG signaling, as shown in Table 5. The current diagnosis through the features of nonlinear phase space is mainly for specific diseases including AFIB and MI, which is usually due to the fact that the features of the previously studied methods are highly sensitive to the diagnosis of abnormalities, however, the discriminatory ability between different disease types is more general. When multiple specific signal types are used for diagnosis and classification, the use of multi-lead signals needs to be introduced to improve the overall diagnostic accuracy and reliability. Although the multi-signal diagnosis proposed in this paper is not as capable as previous studies of single-signal classification, the method directly improves the overall performance in specific application scenarios without over-reliance on other clinical and a priori information on the premise of using only the I-lead ECG signal.

**Table 5:** Performance comparison metrics of the proposed method with the previous study for CVDs detection based on nonlinear characterization methods.

Work	ECG type	CVDs type	Features	Classifier	Classifier			
					F1 (%)	Sen (%)	Spe (%)	ACC (%)
This study	I-lead	NSR, AFIB, AF, ST, SB, RBBB	PSAR	SE-ResNet	94	NA	95.6	NA
Zeng et al. [61]	12 leads	NSR, PVC, PB, LBBB, RBBB	Extracted through VMD, SEE, PSR and ED	Dynamical estimators consisting of constant RBF neural networks	NA	96.81	99.20	98.72
Deng et al. [62]	12 leads	MI	Extracted through VCG	MLP	NA	98.6	97.4	98.4
Nguyen et al. [63]	Single-lead	AFIB	Poincaré plot	2D-CNN	NA	96.82	98.86	98.08
Bartłomiej [11]	Single-lead	AFIB	Spectrogram, scalogram, attractor	2D-CNN	93; 94;**89**	NA	NA	95; 94;**89**
Ma et al. [64]	12 leads	MI	Extracted through cross-clustering coefficient entropy	SVM	NA	NA	NA	95.8

MI, Myocardial Infraction; NA, not applicable; Sen, sensitivity; Spe, specificity; ACC, accuracy; F1, F1-scores; VDM, variational mode decomposition; PSR, phase space reconstruction; SEE, Shannon energy envelope; ED, Euclidean distance; VCG, vectorcardiogram.

### 4.5 Diagnosis of I-lead OSAS

The signal sampling frequency of the original QSAS dataset was 80 Hz, and the processing of up-sampling frequency to 500 Hz was added to the original signal preprocessing method. Periodic reconstruction of attractors was carried out on 80 Hz and 500 Hz data respectively, and the signal diagnostic analysis was carried out by SE-ResNet, the results of which are shown in Table S3, indicating that the sampling frequency will have a significant effect on the attractor reconstruction signal. Therefore, sampling the target signal to the same or similar frequency is a necessary operation.

**Fig. 10:**
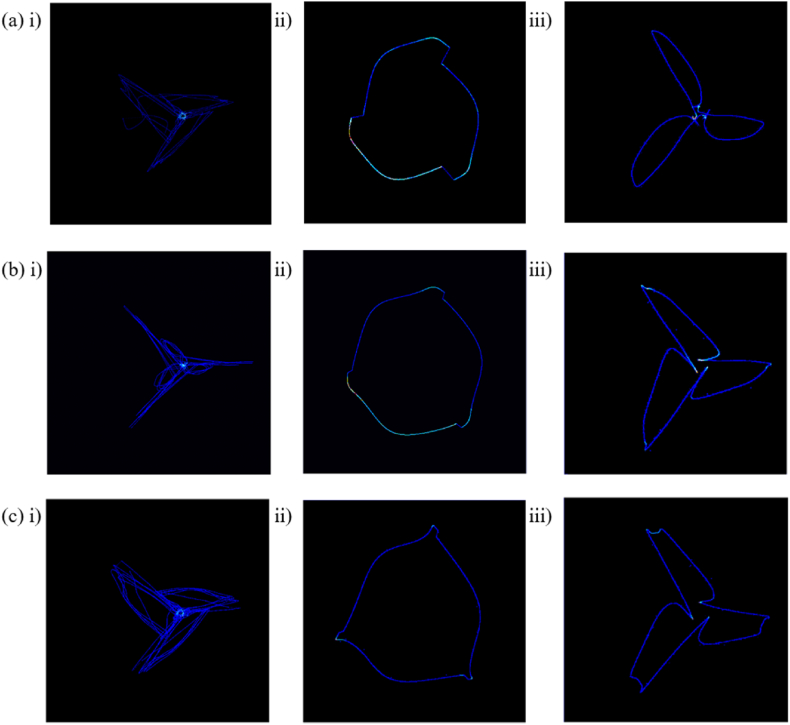
PSAR density map of OSAS. (a) Apnea-obstructive, i) R–R Split, ii) QRS Split, iii) STPQ Split, (b) Apnea-central, i) R–R Split, ii) QRS Split, iii) STPQ Split, (c)Apnea-mixed, i) R–R Split, ii) QRS Split, iii) STPQ Split.

Based on the dataset recording, OSAS was further divided and we extracted the constructs of apnea-obstructive, apnea-central and apnea-mixed categories for APNEA diagnostic data. Based on our proposed PSAR method, the feature density is plotted as shown in Fig. 10, where the results using three different split methods are shown separately. Apnea exhibits a top-out fan-blade shape in the R–R split, which is evident in apnea-obstructive and apnea-mixed, respectively, with the fan-blade shape of apnea-central being more centrally focused. In the Q-R-S split, the three types of apnea signals behave similarly and present a consistent overall figure, their motion behavior during filling and contraction of the ventricle is similar. In the S-TP-Q split, apnea-obstructive and apnea-central exhibit two approximate fan-blade shapes and show a more chaotic state in apnea-mixed after mixing. The tendency of mixing the two apnea signals can be observed from Fig. 10 (c).

For the APNEA diagnosis, we chose NSR and potentially AFIB and APNEA during sleep as the comparison diagnostic groups. Also using SE-ResNet as a diagnostic model, the signal duration was chosen to be 15–30 s to cover continuous apnea features, considering the state during sleep. The diagnostic results are shown in Table 6. Among them, the F1 score and specificity rate, suggest that the proposed cycle reconstruction attractor feature can accurately monitor the appearance of OSAS by I-lead ECG signal in sleep state kind. And R–R split and S-TP-Q split performed better. Diagnostic accuracy confusion matrix of PSAR for OSAS of I-lead ECG signals are shown in Supplementary Material in Fig. S5.

**Table 6:** The diagnosis results of APNEA with sample rate 500 Hz.

Signal	R–R Split		Q-R-S Split		S-TP-Q Split	
	Specificity	F1	Specificity	F1	Specificity	F1
**Normal**	**0.997**	**0.991**	**0.971**	**0.98**	**0.982**	**0.945**
**AFIB**	**0.997**	**0.982**	**0.957**	**0.942**	**0.954**	**0.927**
**Apnea**	**1.0**	**1.0**	**1.0**	**1.0**	**0.989**	**0.992**

We compared other studies on the diagnosis of OSAS by ECG signaling, as shown in Table 7. Previous researchers usually choose the time-domain features and raw ECG signals directly as the input signals of the classifier, the method proposed in this paper shows that PSAR can be more accurate on the OSAS classification task and does not compute the task does not increase significantly.

**Table 7:** Performance comparison metrics of the proposed method with the previous study for OSAS detection based on single-lead ECG signals.

Work	Features	Classifier	Performance			
			AUC	Sen	Spe	Other
This study	PSAR	SE-ResNet	NA	NA	0.922–1.0	F1: 0.992–1.0
Nabanita et al. [65]	SSA & NMF based features	Ensemble Classifier	NA	0.913–0.944	0.933–0.957	ACC: 0.934–0.944
Li T. et al. [66]	Extracted through TQWT, VMD, PSR and ED	Dynamical estimators consisting of neural networks	NA	0.977	0.986	ACC: 0.983
Praveen et al. [67]	Raw ECG	E-DBN	0.960	1.0	0.917	ACC: 0.943
Urtnasan E. et al. [68]	Raw ECG	CNN	0.93	NA	NA	F1: 0.86 ACC: 0.85
Hang L. et al. [69]	Raw ECG	CNN-Transformer	0.882	0.785	0.941	NA
Mahsa B. et al. [70]	R–R intervals and R-peak amplitude	CNN + BiLSTM	0.881	0.815	0.923	F1: 0.84

AUC, area under the receiver operating characteristics curve; Sen, sensitivity; Spe, specificity; ACC, accuracy; NA, not applicable; SSA, singular spectrum analysis; NMF, non-negative matrix factorization; TQWT, wavelet transform; VMD, variational mode decomposition; PSR, phase space of three-dimension; ED, Euclidian distance; E-DBN, enhanced-deep belief networks.

## 5 Conclusion

This study provides a method to analyze ECG signals based on PSAR, which is combined with machine learning to realize accurate and rapid diagnosis of cardiovascular diseases. We established the attractor reconstruction characterization method for cycle splitting based on the cardiac cycle signal characteristics, where the splitting cycles include R–R split, Q-R-S split, and S-TP-Q split. Morphological features were analyzed and density maps were constructed as machine learning features, which enables an intelligent diagnostic process.

Evaluated the diagnostic effectiveness of PSAR under the SE-ResNet model under the construction of a first-lead equilibrium dataset, the diagnostic effectiveness of the full-period signals generally exceeds 90 %, and the health state recognition rate is close to 100 %. After the signal split, the diagnostic accuracy of Q-R-S split signals for RBBB increased by 3 %. Among the S-TP-Q signal split signals, SB and ST had the highest detection success rate. The PSAR results show that there is a clearer distinction between the manifestations of different split methods in different CVDs.

In addition, we similarly validated the diagnostic effect of APNEA signaling on I-lead data, and showed that PSAR exhibited good performance for precisely recorded sleep state signaling. As a result, the signal diagnosis of typical signals in this region is correspondingly improved.

In the future, we will develop a set of visual intelligent diagnostic analysis tools based on PSAR, which will be deployed in portable and wearable health monitoring devices to provide quick diagnostic tools for CVDs and health monitoring.

## Data availability

Data will be made available on request.

## CRediT authorship contribution statement

**Ze Zhang:** Writing – original draft, Visualization, Validation, Methodology, Investigation, Formal analysis, Data curation, Conceptualization. **Kayo Hirose:** Writing – review & editing, Validation, Methodology, Formal analysis, Data curation, Conceptualization. **Katsunori Yamada:** Writing – review & editing, Visualization, Supervision, Formal analysis. **Daisuke Sato:** Writing – review & editing, Visualization, Supervision, Methodology. **Kanji Uchida:** Writing – review & editing, Validation, Supervision. **Shinjiro Umezu:** Writing – review & editing, Visualization, Supervision, Resources, Project administration, Methodology, Investigation, Funding acquisition, Conceptualization.

## Declaration of competing interest

The authors declare that they have no known competing financial interests or personal relationships that could have appeared to influence the work reported in this paper.
