# Supplementary material for: A periodic split attractor reconstruction method facilitates cardiovascular signal diagnoses and obstructive sleep apnea syndrome monitoring
Source: Heliyon. 2024 Aug 3;10(15):e35623. doi: 10.1016/j.heliyon.2024.e35623 (PMC11337694; doi:10.1016/j.heliyon.2024.e35623)
Supplement: Multimedia component 1 [file mmc1.docx]

**Supplementary material**

**A periodic split attractor reconstruction method facilitates cardiovascular signal diagnoses and obstructive sleep apnea syndrome monitoring**

Ze Zhang^1,a^, Kayo Hirose^2,a^, Katsunori Yamada^3^, Daisuke Sato^4^, Kanji Uchida^2^, Shinjiro Umezu^1*^

*^1^Graduate School of Creative Science and Engineering, Department of Modern Mechanical Engineering, Waseda University, 3-4-1 Okubo, Shinjuku-ku, Tokyo 169-8555, Japan*

*^2^* *Department of Anesthesiology and Pain Relief Center, The University of Tokyo Hospital, 7-3-1 Hongo, Bunkyo-ku, Tokyo 113-8655, Japan*

*^3^Faculty of Economics, Kindai University, 228-3 Shin-Kami-Kosaka, Higashi-Osaka 577-0813, Japan*

*^4^Department of Pharmacology, University of California, Davis, Genome Building Rm3503, Davis, CA 95616–8636, USA*

**^a)^ These authors equally contributed to this work.**

***Corresponding Authors:**

**Shinjiro Umezu, Professor, E-mail: umeshin@waseda.jp**

# S.1 Wavelet transform and wave group extraction

Further extracting peak positions based on decomposed signals. First, get the R-wave. This waveform is the most characteristic peak and is easy to detect. Using the wavelet transform used in the previous section, the ECG data of only D3, D4, and D5 elements of the frequency characteristics are taken out. This is the frequency band in which the R-wave is most characteristic. From this data, the function value is determined and the R-wave is obtained. Based on the references, the value is the maximum value of the data for D3, D4, and D5 multiplied by 0.15. After detecting the part that exceeds the function value, the maximum value is taken at the interval of 160ms before and after the detection, and it is taken as the point of the R-peak. Then, slide the 200ms data and repeat the detection according to the corresponding value . Starting from the R-peak obtained in this procedure, other waveforms are also extracted. Q-wave and S-wave detect the peak before and after the extracted R-wave. Check the adjacent points in order, and extract the points where the slope became negative. Then, when the difference with the R-wave is less than or equal to the function value, it is judged as the peak caused by noise, and it is repeated to the adjacent point. Then the P-wave and the T-wave are extracted. Take a window in the range of 120ms from the obtained Q-wave, and set the maximum value point as the P-wave. Take a window in the range of 120ms interval from the obtained S-wave, and set the maximum point as the T-wave. Each resulting point is compared to whether it is larger than the adjacent point, and if it is smaller, it is replaced by a point where the slope is positive.

**
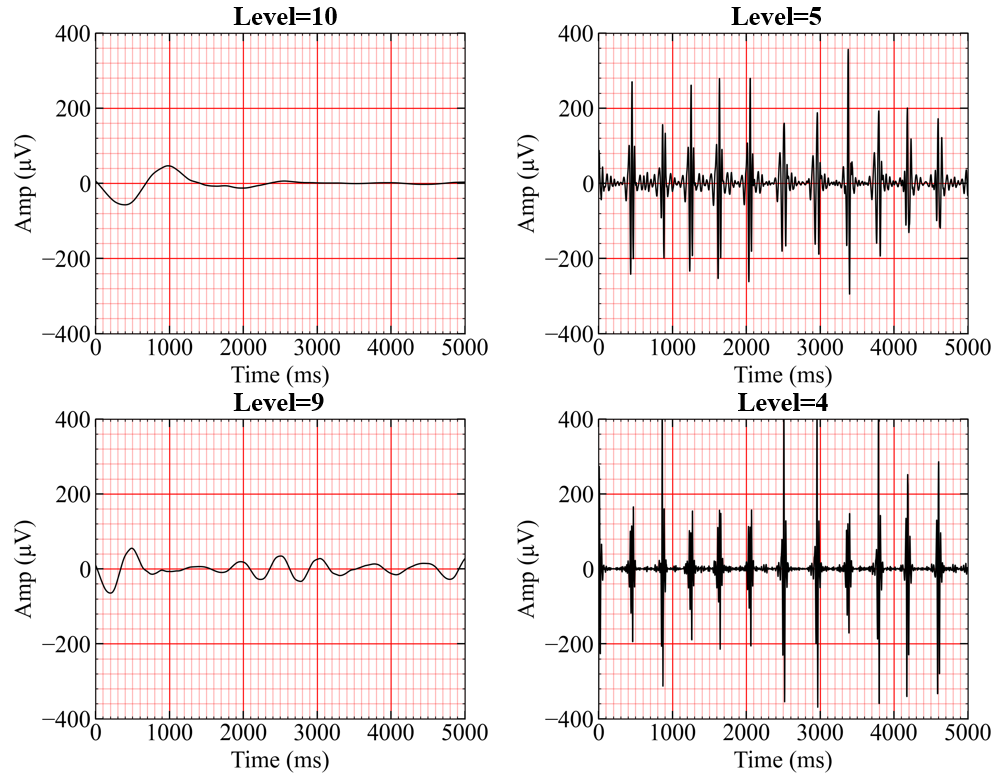

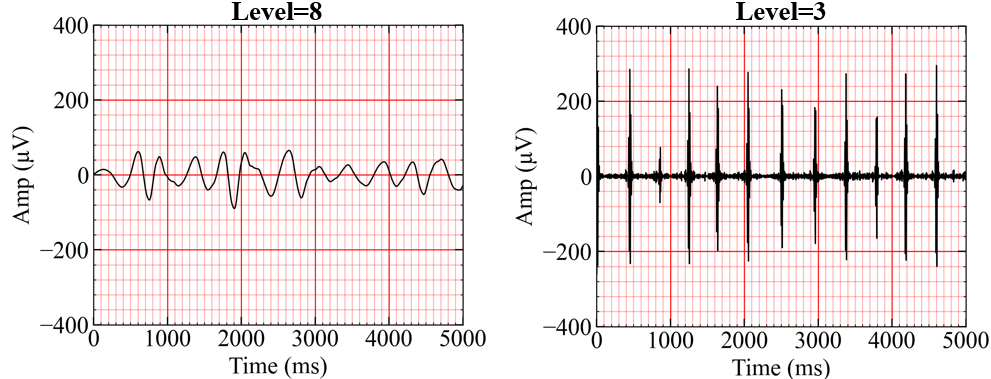
**

**
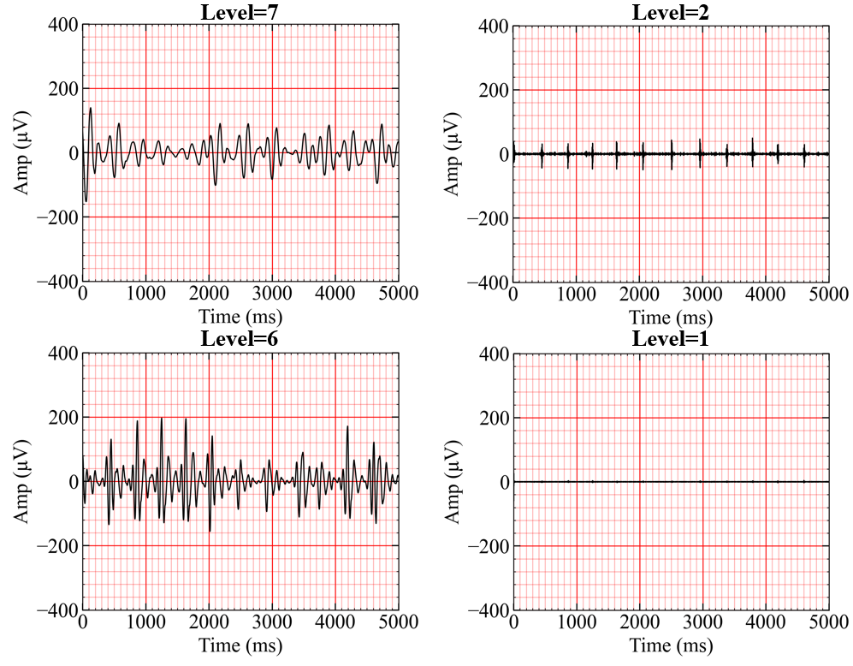
**

**Fig. S1** 10-level DWT decomposition signal.

Perform discrete wavelet decomposition on the ECG signal, and the 10 level decomposition results are shown in Fig. S1.

# S.2 PSAR with different delay of ideal signal

**
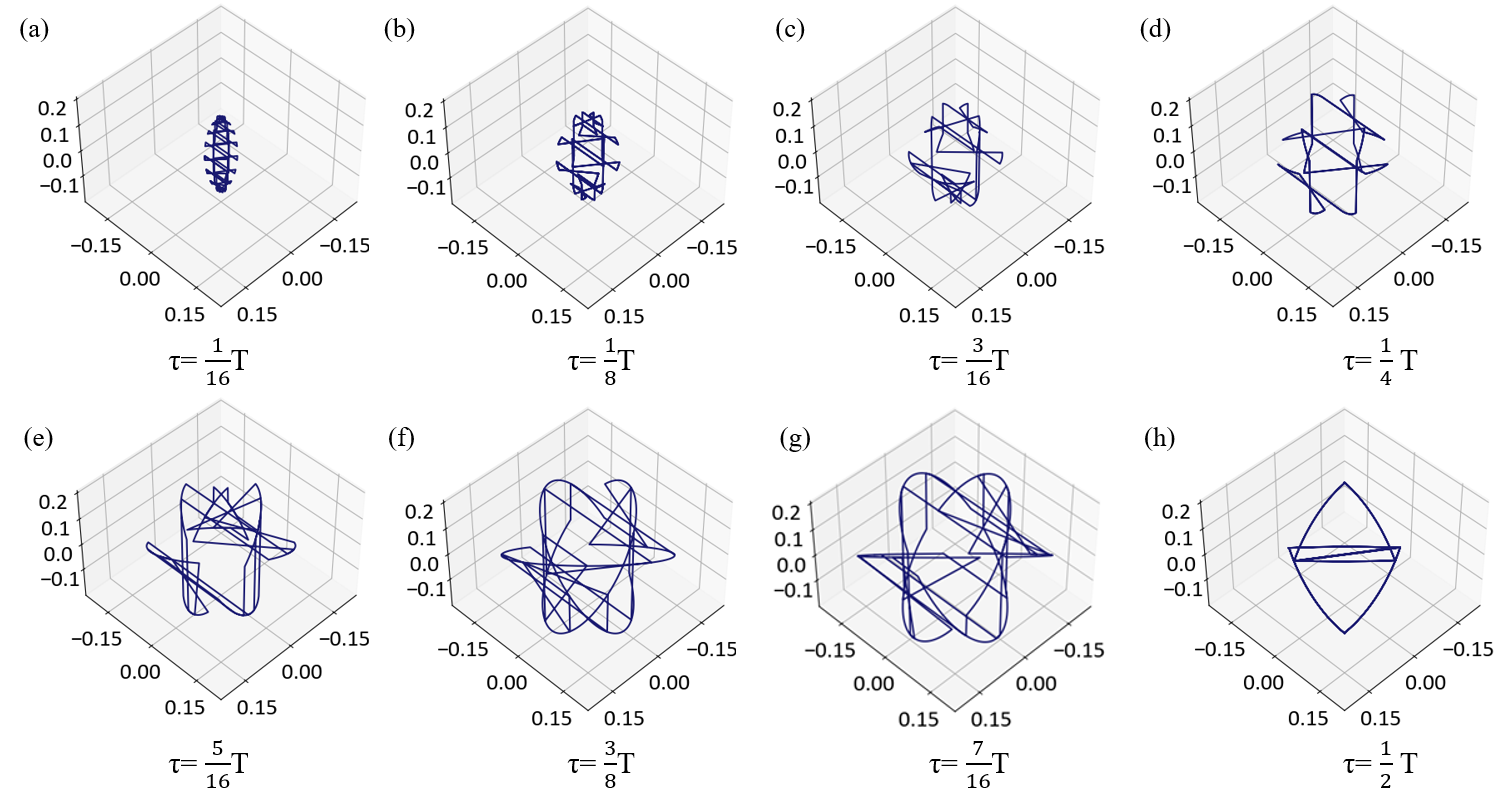
**

**
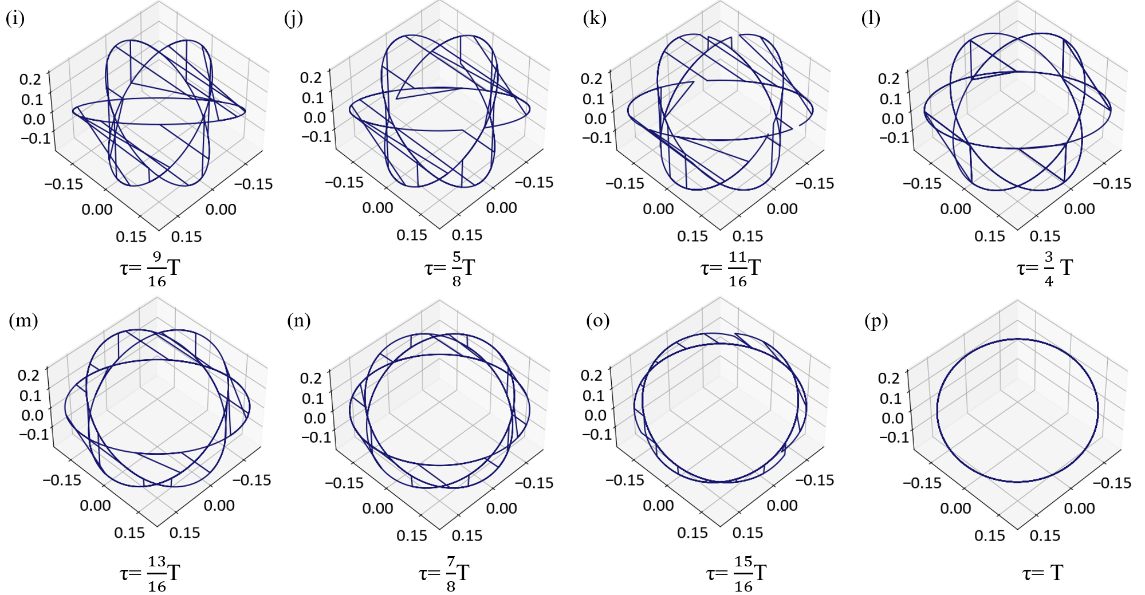
**

**Fig. S2** Sinusoidal function signal (period $T$) with different delays *τ*$\in[0,T]$ reconstruction result of attractor.

**
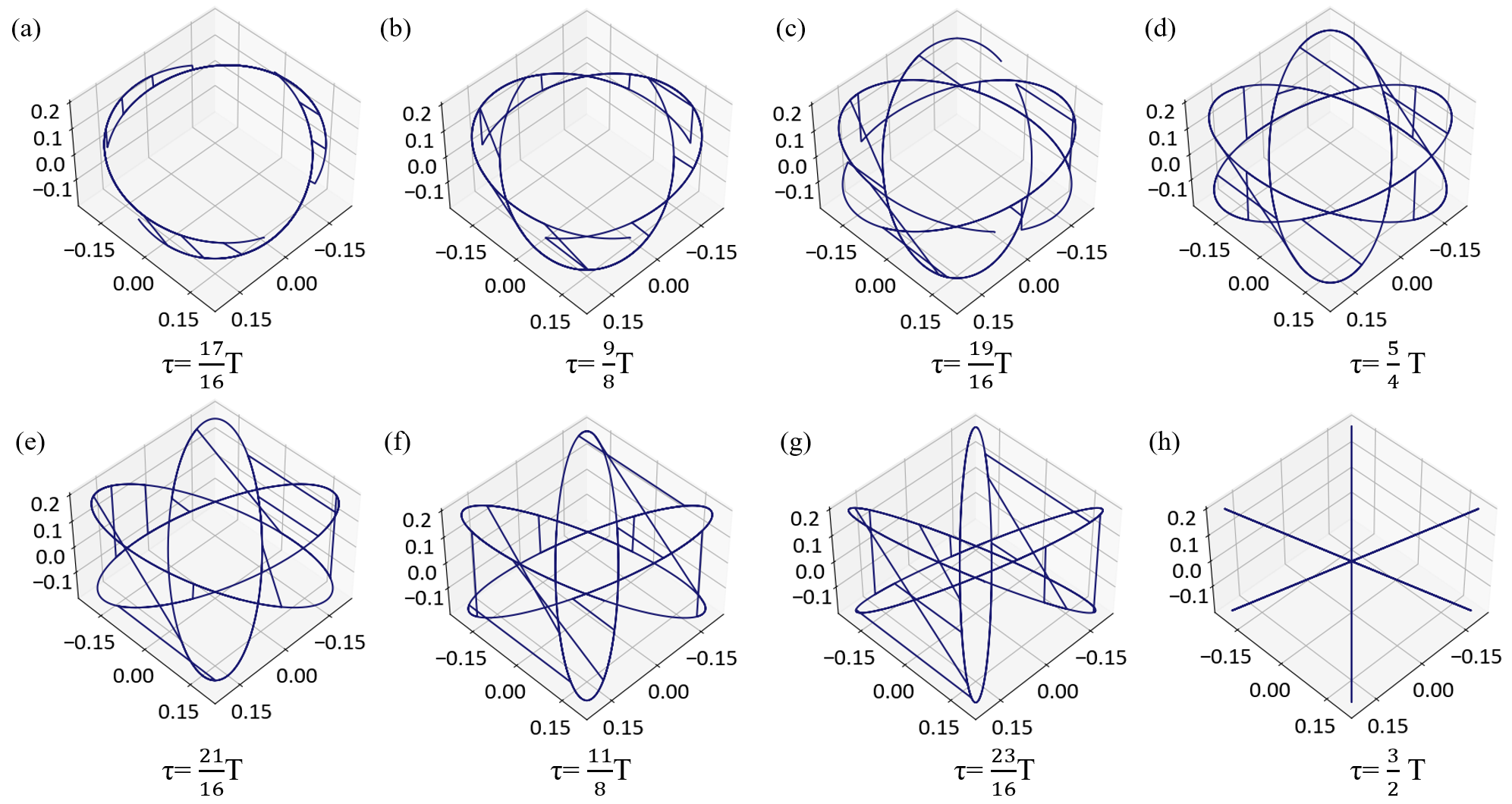
**

**
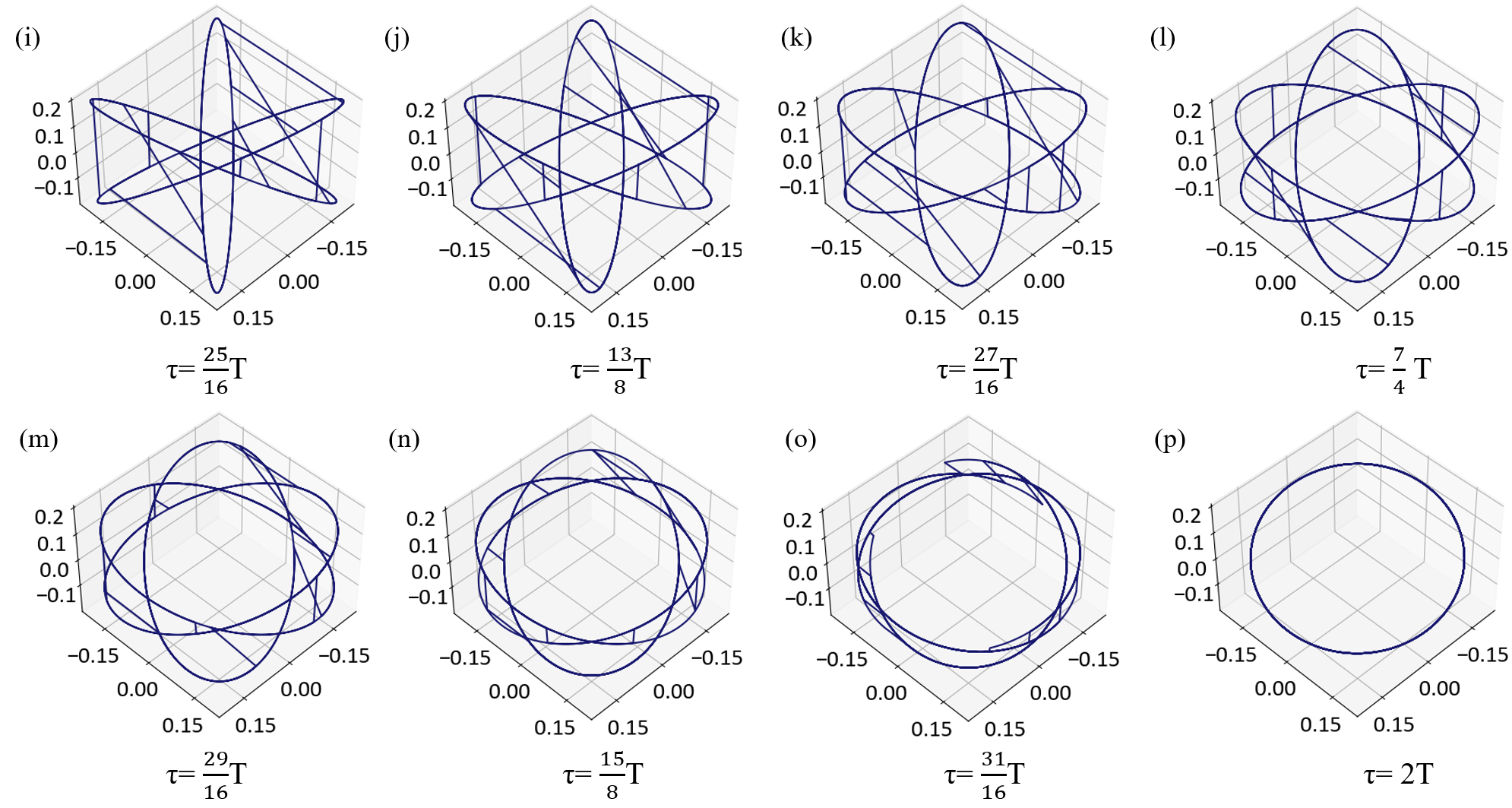
**

**Fig. S3** Sinusoidal function signal (period $T$) with different delays *τ*$\in[T,2T]$ reconstruction result of attractor.

Fig. S2 and Fig. S3 respectively show the results of reconstruction of Attractor under ideal signal for delay parameters with 16 equal periods and periods of [0, T] and [T, 2T]. We can observe that the overall trend of attractor reconstruction is the same under the adjacent *τ*. This shows that within the complex ECG wave group, the limited delay parameter fluctuation has limited influence on the overall signal reconstruction attractor. Finally, the attractor density map result of ECG signal reconstruction for a period of time can be input into the machine learning model as an effective and accurate feature for training and intelligent diagnosis

# S.3 Diagnostic efficacy of PSAR

This section complements the results of PSAR in the diagnosis of CVDs and OSAS. It includes the precision confusion matrix, and the speed of PSAR running on the test GPUs.


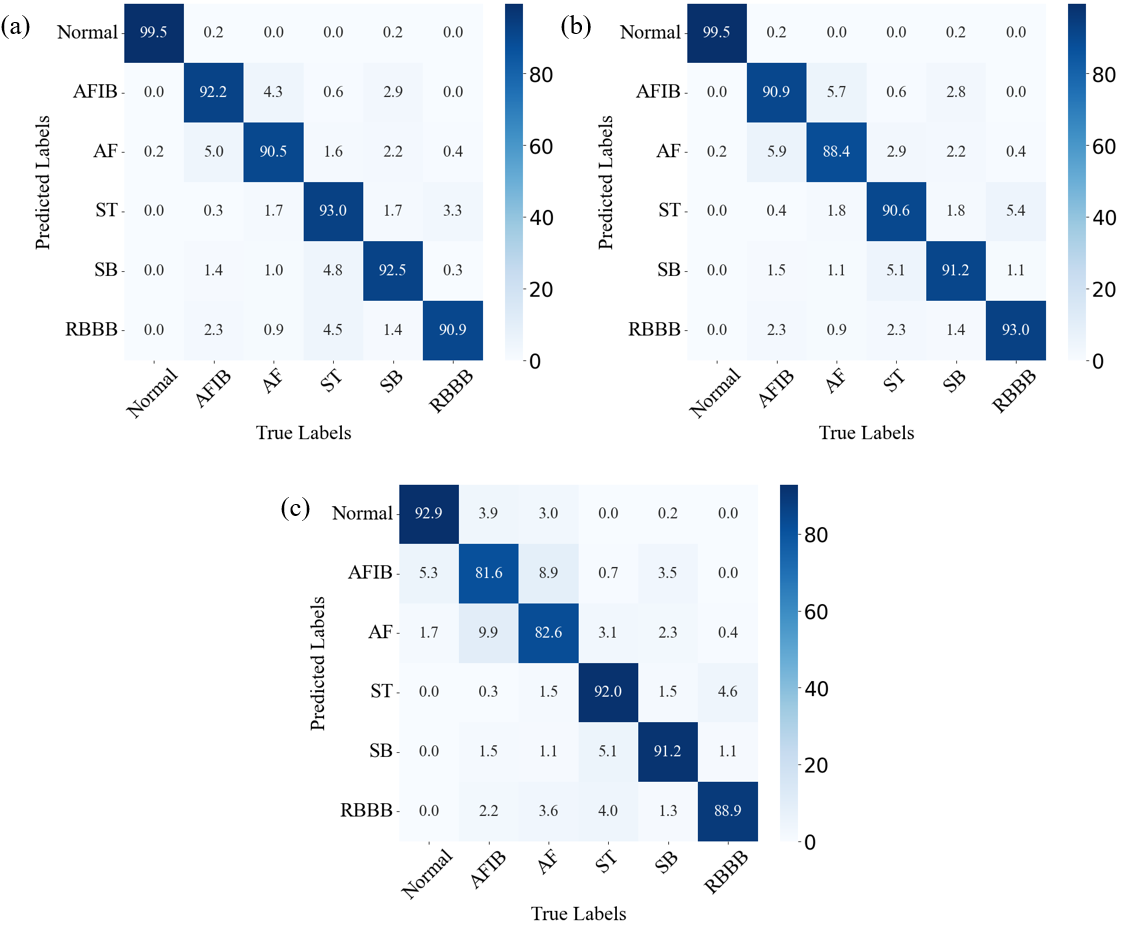


**Fig. S4** Diagnostic accuracy confusion matrix of PSAR for CVDs of I-lead ECG signals. (a) R-R split, (b) Q-R-S split, (c) S-TP-Q split.

**
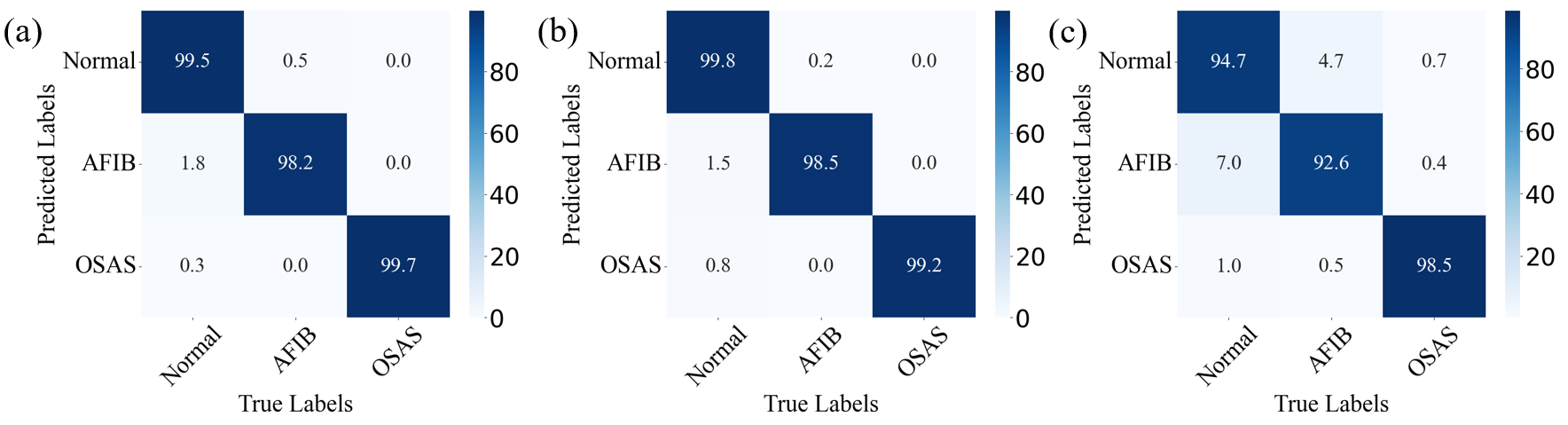
**

**Fig. S5** Diagnostic accuracy confusion matrix of PSAR for OSAS of I-lead ECG signals. (a) R-R split, (b) Q-R-S split, (c) S-TP-Q split.

**Table S1**

**Diagnostic speed comparison (attractor data set).**

| **Layers in Model** | **Time (ms) per inference step** | | | | | |
| --- | --- | --- | --- | --- | --- | --- |
|  | **CPU*** | **GPU*** | **CPU(SE)*** | **GPU(SE)*** | **CPU(Keras)** | **GPU(Keras)** |
| 18 | 55.9 | 41.7 | 54.7 | 41.2 | - | - |
| 34 | 70.2 | 39.0 | 70.8 | 38.4 | - | - |
| 50 | 99.9 | 38.5 | 100.1 | 37.7 | 58.2 | 4.6 |
| 101 | 158.3 | 43.7 | 157.6 | 42.5 | 89.6 | 5.2 |
| 152 | 205.2 | 43.6 | 203.1 | 44.6 | 127.4 | 6.5 |

*It represents the standard ResNet measuring speed on the attractor data set, (SE) * represents the SE-ResNet measuring speed on the attractor data set, and (Keras) represents the model budget speed released by the Keras platform[42].

**Table S2**

**The diagnosis results of attractor reconstruction of OSAS at 80Hz sample rate.**

| **Signal type** | **Evaluation** | | | |
| --- | --- | --- | --- | --- |
|  | **Precision** | **Recall** | **Specificity** | **F1** |
| **Normal** | **0.903** | **1.0** | **0.951** | **0.949** |
| **AFIB** | **1.0** | **0.791** | **1.0** | **0.883** |
| **Apnea** | **1.0** | **1.0** | **1.0** | **1.0** |

**Table S3**

**The classification results of attractor reconstruction of OSAS at different sample rate.**

| **Signal type** | **Evaluation** | | | |
| --- | --- | --- | --- | --- |
|  | **Precision** | **Recall** | **Specificity** | **F1** |
| **Apnea-80Hz** | **1.0** | **1.0** | **1.0** | **1.0** |
| **Apnea-500Hz** | **1.0** | **1.0** | **1.0** | **1.0** |

# Reference

1. Sahoo, S., et al., *Multiresolution wavelet transform based feature extraction and ECG classification to detect cardiac abnormalities.* Measurement, 2017. **108**: p. 55-66.
